# Supplementary material for: Prevalence of Cow’s Milk Allergy in Infants from an Urban, Low-Income Population in Chile: A Cohort Study
Source: Nutrients. 2025 May 29;17(11):1859. doi: 10.3390/nu17111859 (PMC12157014; doi:10.3390/nu17111859)
Supplement: Supplementary file 1 [file nutrients-17-01859-s001.zip › nutrients-3652189-supplementary.pdf]

**Table S1.** Symptom checklist used by the study nurse during monthly follow-up. Infants presenting with ≥2 symptoms were referred for evaluation by a pediatric gastroenterologist.

**Does the child currently present the following symptoms?**

| **Gastrointestinal (GI) Symptom** | **Yes** | **No** |
|---|---|---|
| -Frequent regurgitations (5 or more times/day) | ☐ | ☐ |
| -Vomiting (2 or more times/day) | ☐ | ☐ |
| -Colic (≥3 hrs/day, ≥3 days/week) | ☐ | ☐ |
| -Feeding refusal (≥2 feedings/day) | ☐ | ☐ |
| -Diarrhea (change in bowel habits) | ☐ | ☐ |
| -Mucus in stools | ☐ | ☐ |
| -Bloody stools | ☐ | ☐ |
| -Constipation | ☐ | ☐ |
| -Abdominal distension | ☐ | ☐ |
| -Poor weight/height gain (weight or height <-1SD for age ) | ☐ | ☐ |
| -Anemia (per hematocrit-hemoglobin chart) | ☐ | ☐ |
| -Suspected allergic enteropathy/colitis | ☐ | ☐ |
| -Irritability (≥3 days/week or >3 weeks duration) | ☐ | ☐ |

| **Dermatological Symptoms** | **Yes** | **No** |
|---|---|---|
| -Atopic dermatitis (≥2 of the following: erythematous eczematous plaques, excoriations, erythematous papules, scaling patches, dry-rough skin)<br>-Eyelid/lip edema<br>-Urticaria<br>-Perianal erythema | ☐ | ☐ |

| **Respiratory Symptom (not associated with another condition/infection)** | **Yes** | **No** |
|---|---|---|
| -Rhinorrhea | ☐ | ☐ |
| -Cough | ☐ | ☐ |
| -Wheezing | ☐ | ☐ |
| -Laryngeal edema/dysphonia | ☐ | ☐ |
| -Bronchial obstruction | ☐ | ☐ |
| -Anaphylactic shock | ☐ | ☐ |

**Table S2.** Number of lost cases and percentage of retention during a one year follow-up in a cohort of 552 infants.

| Month of follow-up | Loss of cases (accumulated number) | Retention (%) |
|---|---|---|
| 1 | 0 | 100.0 |
| 2 | 22 | 96.0 |
| 3 | 38 | 93.1 |
| 4 | 45 | 91.8 |
| 5 | 52 | 90.5 |
| 6 | 54 | 90.2 |
| 7 | 60 | 89.1 |
| 8 | 64 | 88.4 |
| 9 | 74 | 86.5 |
| 10 | 82 | 85.1 |
| 11 | 92 | 83.3 |
| 12 | 109 | 80.2 |

**Table S3.** Diagnosis of patients derived for gastroenterology evaluation, not fulfilling criteria for CMA.

| Diagnosis | Frequency (Total = 90) | % |
|---|---|---|
| Healthy | 19 | 21 |
| Physiologic regurgitation | 20 | 22 |
| Primary gastroesophageal reflux disease | 6 | 7 |
| Infant colics | 6 | 7 |
| Acute gastroenteritis | 5 | 5.5 |
| Functional constipation | 7 | 8 |
| Isolated rectorrhagia | 1 | 2 |
| Atopic dermatitis | 8 | 9 |
| Other dermatitis | 6 | 7 |
| Underfeeding | 6 | 7.5 |
| Other diagnosis | 3 | 4 |
